# Factors favouring the evolution of multidrug resistance in bacteria

**DOI:** 10.1098/rsif.2020.0105

**Published:** 2020-07-29

**Authors:** Eliott Jacopin, Sonja Lehtinen, Florence Débarre, François Blanquart

**Affiliations:** 1 Centre for Interdisciplinary Research in Biology (CIRB), Collège de France, CNRS, INSERM, PSL Research University, Paris, France; 2 AgroParisTech, Université Paris-Saclay, Paris, France; 3 The Oxford Big Data Institute, Nuffield Department of Medicine, University of Oxford, Oxford, UK; 4 Institute of Integrative Biology, Department of Environmental Systems Science, ETH Zurich, Zurich, Switzerland; 5 Sorbonne Université, CNRS, Université Paris Est Créteil, Université de Paris, INRAE, IRD, Institute of Ecology and Environmental sciences of Paris, iEES-Paris (UMR 7618), 75005 Paris, France; 6 Infection Antimicrobials Modelling Evolution, UMR 1137, INSERM, Université de Paris, Paris, France

**Keywords:** antimicrobial resistance, multidrug resistance, mathematical modelling, adaptation

## Abstract

The evolution of multidrug antibiotic resistance in commensal bacteria is an important public health concern. Commensal bacteria such as *Escherichia coli*, *Streptococcus pneumoniae* or *Staphylococcus aureus*, are also opportunistic pathogens causing a large fraction of the community-acquired and hospital-acquired bacterial infections. Multidrug resistance (MDR) makes these infections harder to treat with antibiotics and may thus cause substantial additional morbidity and mortality. Here, we develop an evolutionary epidemiology model to identify the factors favouring the evolution of MDR in commensal bacteria. The model describes the evolution of antibiotic resistance in a commensal bacterial species evolving in a host population subjected to multiple antibiotic treatments. We combine statistical analysis of a large number of simulations and mathematical analysis to understand the model behaviour. We find that MDR evolves more readily when it is less costly than expected from the combinations of single resistances (positive epistasis). MDR frequently evolves when bacteria are in contact with multiple drugs prescribed in the host population, even if individual hosts are only treated with a single drug at a time. MDR is favoured when the host population is structured in different classes that vary in their rates of antibiotic treatment. However, under most circumstances, recombination between loci involved in resistance does not meaningfully affect the equilibrium frequency of MDR. Together, these results suggest that MDR is a frequent evolutionary outcome in commensal bacteria that encounter the variety of antibiotics prescribed in the host population. A better characterization of the variability in antibiotic use across the host population (e.g. across age classes or geographical location) would help predict which MDR genotypes will most readily evolve.

## 1. Introduction

The emergence of antibiotic-resistant bacterial strains is a public health problem. Infections by antibiotic-resistant strains result in longer duration of hospital stay, poorer prognosis and additional costs compared with infections by sensitive strains [1–9]. Multidrug resistance (MDR) is even more critical, as it may be associated with an even higher risk of inappropriate therapy and a longer time to appropriate treatment than single resistances [10–13]. In spite of the clinical importance of MDR, there are much fewer models investigating the factors favouring the evolution of MDR than the evolution of single resistance.

It is useful to distinguish between the factors that explain the *origin* of MDR and those that explain their *proliferation* [14]. MDR strains can originate because a single biological mechanism confers resistance to multiple drugs, because several genes conferring resistances to several antibiotics are genetically linked together on the chromosome or a plasmid, or because multiple mutations conferring resistance to multiple antibiotics have evolved over the course of treatment in a host. This latter explanation may explain MDR in bacterial species causing chronic infections such as *Mycobacterium tuberculosis* [15]. Here, we focus on the evolutionary forces that promote the proliferation of MDR strains once they have originated. Three hypotheses have been proposed for MDR strain proliferation [14]:

(i) When resistance is associated with a fitness cost, the overall cost of MDR may be lower than predicted by combining the costs of individual resistances, which would favour the evolution of MDR over single resistances.
(ii) The use of multiple antibiotics in the host population may favour MDR. In particular, many bacterial species are mainly commensal, and thus primarily in contact with antibiotics not because they caused an infection themselves, but because their host is treated for infections caused by other species: this phenomenon is called ‘bystander selection’ [16]. These species would experience occasional antibiotic selection by the full diversity of antibiotics prescribed in the host population, which may select for MDR.
(iii) If the host population is structured in different classes taking antibiotics at different rates, MDR may be over-represented at the whole population level because MDR strains are favoured in the classes with high rates of antibiotic treatments.

Until recently, few theoretical studies had examined these three hypotheses for the proliferation of MDR. Lehtinen *et al*. [17] developed an epidemiological model to describe the evolution of MDR when the host population is structured (e.g. different age classes or geographical regions). This study found that MDR is over-represented when two criteria are met: firstly, variation in antibiotic consumption rates across host classes is correlated for different antibiotics (‘aligned use’—classes with high consumption of one antibiotic also consume other antibiotics at a high rate); and secondly, the classes are strongly assortatively mixing: transmission within classes is much more frequent than transmission between classes.

The structure of the host population may thus play a central role in the evolution of MDR but some questions remain unanswered. First of all, many previous models for the evolution of resistance do not explicitly consider hosts that are under treatment. These models assume that treatment either does not affect resistant bacteria at all, and that treatment instantaneously removes all sensitive bacteria from the host and is stopped. Yet, treated hosts form a protected niche in which resistant strains preferentially replicate, which can favour the coexistence of both resistant and sensitive strains [18]. Secondly, in a structured host population, which resistance genotypes evolve must depend on the interaction between the rate of transmission across different classes (mixing), and how the use of different antibiotics varies across classes. Thirdly, recombination between resistance loci should also affect the evolution of MDR. This is true both in the context of a single host population and when the host population is structured. In a structured host population, mixing and recombination of different genotypes from distinct classes may create maladapted genotypes that are distinct from those that would be favoured in the absence of recombination. It is not clear, in these more general scenarios of heterogeneity in antibiotic use, variable mixing and recombination, which genotypes will evolve. Lehtinen *et al*.'s study explored the role of non-aligned variation in antibiotic use, mixing and recombination, in separate extensions of their main model. But, to our knowledge, no study has examined the more general scenario with explicit treatment, arbitrary variations in antibiotic use and level of transmission between classes, and recombination.

Here, we develop a model examining the evolution of MDR in a structured host population that complements existing models in these aspects: we explicitly model the dynamics of untreated and treated individuals, in contrast with previous models often assuming that treatment instantaneously clears individuals colonized by a sensitive strain, and does not affect individuals colonized by a resistant strain. We model recombination between different strains, and finally, we extend this model to a host population structured in different classes and we examine how the variation in antibiotic use, the rate of transmission between classes and recombination affect MDR evolution.

## 2. Methods

### 2.1. The model

#### 2.1.1. General features of the model

We model the evolution of antibiotic resistance using a compartmental model describing the dynamics of different types of hosts. We assume that the overall host population size is stable (scaled to one without loss of generality) and we describe the dynamics of the frequency of hosts colonized by the bacterial strain *i*, under treatment *m*, denoted by *X_i_*_,*m*_. We define $R^{*}$ the set of all possible bacterial strains, and R the set of all possible colonization states of the hosts, $R=R^{*}\cup \emptyset$ where the empty set ∅ denotes the absence of colonization. We denote by $T^{*}$ the set of possible antibiotic treatments ($m\in T^{*}$) and by $T=T^{*}\cup \emptyset$ the set of all possible host treatment status, including no treatment. A treatment can be a combination of multiple drugs.

The following events change the frequencies of the different host types: colonization of uncolonized hosts by a bacterial strain (transmission), clearance of colonized hosts, antibiotic treatment and supercolonization. Supercolonization describes the colonization by a challenger strain of a host already colonized by a resident strain.

We assume that the bacterial species has a mainly commensal lifestyle. Therefore, we do not model the occasional infection by the focal species, but only its asymptomatic (commensal) carriage by hosts. This assumption corresponds to some common species (e.g. *Escherichia coli*, *Streptococcus pneumoniae*, *Staphylococcus aureus*). We also assume that *de novo* evolution of resistance is negligible: resistance is *primary*, i.e. caused by resistant strains already circulating in the population.

#### 2.1.2. Colonization of uncolonized hosts

An uncolonized host is colonized at a rate *β_i_*_,*m*_*X_i_*_,*m*_ by a strain of genotype *i* in a host under treatment *m*. The parameter *β_i_*_,*m*_ is the transmission rate of the strain *i* carried by a host under treatment *m* (all parameters are summarized in table 1 below). Transmission is reduced by the cost of resistance [19,27] and reduced by effective treatment. That is, *β_i_*_,*m*_ = *β*(1 − *χ_i_*)(1 − *ψ_i_*_,*m*_) where *β* is the baseline transmission rate, *χ_i_* is the transmission cost of resistance of strain *i* (equal to 0 when the strain is fully sensitive) and *ψ_i_*_,*m*_ is the effect of treatment *m* on the transmission rate (equal to 0 when the strain is resistant to treatment *m*). For a given genotype, the reduction in transmission due to the cost, 1 − *χ_i_*, is computed by multiplying the effects of individual resistances that the genotype carries and pairwise interactions between all pairs of resistances. This introduces *cost epistasis*, whereby the cost of carrying multiple resistance may not be equal to the multiplication of all individual resistances' costs. Negative cost epistasis in particular may favour the evolution of MDR. For example, if there are only two drugs, the reduction in transmission for the double resistant is $1-\chi_{R_{1,2}}=(1-\chi_{R_{1}})(1-\chi_{R_{2}})(1-\chi_{e})$ where *χ_e_* is the transmission cost epistasis parameter. In a similar way, the effect of treatment in reducing transmission 1 − *ψ_i_*_,*m*_ depends on the multiplicative effects of individual drugs present in the treatment and pairwise interactions for all pairs of drugs to which genotype *i* is sensitive [28,29].

**Table 1.** Description of the variables and the parameters. All rates are expressed in units of month^−1^.

variables and notations				
*X_i_*_,*m*_	density of individuals colonized by strain *i* under treatment *m*			
*η_j_*_,*k*,*m*→_*_i_*	probability that a host with strain *j* and treatment *m* invaded by the challenger strain *k* is eventually colonized with strain *i* following recombination and within-host competition			
*γ_i_*_,*m*_	total gains for the strain *i* in hosts under treatment *m* upon supercolonization			
*π_i_*_,*m*_	total losses for the strain *i* in hosts under treatment *m* upon supercolonization			

parameters				
name	meaning	sampling range	sampling method	justification
*β_i_*_,*m*_	transmission rate of strain *i* under treatment *m*	—	—	composed of baseline transmission rate *β*, transmission cost of resistance *χ_i_*, and effect of treatment on transmission *ψ_i_*_,*m*_
*β*	baseline transmission rate	[2, 12]	uniform	tuned to obtain a prevalence ranging from approximately 15% to 95%, corresponding to commensal bacterial species
*χ_i_*	transmission cost of resistance for strain *i* composed of:			transmission costs of resistance largely unknown. *In vitro*, resistance mutations reduce fitness by −30% to 0% [19]. Epistasis assumed to be symmetric around 0 and small
	single resistance costs	[5 × 10^−4^, 0.5]	logarithmic	
	pairwise transmission cost epistasis	[−0.05, 0.05]	uniform	
*ψ_i_*_,*m*_	effect of treatment *m* on the transmission of strain *i*, composed of			effective antibiotic treatment should reduce transmission. Since the magnitude of this effect is unknown, we examine a wide range. Drug interactions assumed to be symmetric around 0 and small
	single drug effects	[0, 1]	uniform	
	pairwise drug interactions	[−0.05, 0.05]	uniform	
*ω_i_*_,*m*_	competitive ability of the strain *i* under treatment *m*	—	—	composed of within-host costs *c_i_* and effect of treatment *ɛ_i_*_,*m*_
*c_i_*	within-host cost of resistance for strain *i* composed of:			within-host costs of resistance largely unknown. The sampling ranges are assumed to be the same as for transmission costs
	single resistance costs	[5 × 10^−4^, 0.5]	logarithmic	
	pairwise within-host cost epistasis	[−0.05, 0.05]	uniform	
*ɛ_i_*_,*m*_	effect of treatment *m* on within-host competitive ability of strain *i,* composed of:			effective antibiotic treatment should reduce within-host competitive ability. Since the magnitude of this effect is unknown, we examine a wide range. Drug interactions assumed to be symmetric around 0 and small
	single drug effects	[0, 1]	uniform	
	pairwise drug interactions	[−0.05, 0.05]	uniform	
$\tau_{T_{1}}$	rate of treatment *T*_1_	[0, 0.2]	uniform	rate of treatment is of the order of 0.1–1 per year across countries [20,21]
$\tau_{T_{2}}$	rate of treatment *T*_2_	[0, 0.2]	uniform	
$\tau_{T_{1,2}}$	rate of combination treatment *T*_1,2_	[2 × 10^−4^, 0.2]	logarithmic	combination treatment is rare. Treatment of common infections relies on single drug therapy [22]
*ν_m_*	rate of end of treatment *m*. 1/*ν_m_* is the mean duration of the antibiotic course	[1, 6]	uniform	an antibiotic course typically lasts for a few days to weeks
*u_i_*	natural clearance rate of strain *i*	[0.1, 3]	uniform (same for all strains)	timescale of clearance of *S. pneumoniae* or *E. coli* is of the order of a few weeks to months [23–25]
*a_i_*_,*m*_	rate of clearance of strain *i* by treatment *m*	set to 20 (when strain *i* is sensitive to treatment *m*)		set to a large enough value to ensure a sensitive strain is cleared during the few days to a week of a typical antibiotic course
*σ*	probability of successful supercolonization	[0, 1]	uniform	unknown, we assume a wide range
*ρ*	probability of recombination during a supercolonization event	[10^−4^, 1]	logarithmic	unknown; assumed to be typically small as many variants conferring resistance appear confined in stable clones [26]

#### 2.1.3. Treatments

The rate of start of treatment with treatment *m* is denoted *τ_m_*, and the rate of treatment stop is denoted *ν_m_*. Thus, 1/*ν_m_* is the expected duration time of the treatment *m*. These rates do not depend on the colonization status of the host. This results from the assumption that the focal species is mainly a commensal: as a consequence, antibiotic treatment is most of the time linked to an infection unrelated to the focal species (‘bystander’ antibiotic selection, [16]).

#### 2.1.4. Clearance

The strain may be cleared from a host, either naturally or by antibiotic treatment. We denote *u_i_* the natural rate of clearance of the strain *i*, and *a_i_*_,*m*_ the rate of clearance of the strain *i* by antibiotic treatment *m*. We assume *a_i_*_,*m*_ = 0 when the strain *i* is resistant to treatment *m* (perfect resistance).

#### 2.1.5. Supercolonization and within-host competition

Supercolonization corresponds to individuals already colonized by a strain *i* under treatment *m* that are colonized by a challenger strain *j* replacing the resident strain *i*. The outcome of supercolonization is decided by within-host competition between the resident and the challenger strain. Supercolonization occurs with a reduced probability compared to transmission to uncolonized hosts. The probability of successful supercolonization is denoted *σ* and is assumed to be the same for all pairs of (resident, challenger) strains.

During supercolonization, recombination and within-host competition occur. Recombination happens with probability *ρ* and may produce new genotypes. Resistance to each drug is determined by a single diallelic locus (sensitive or resistant allele). These genotypes compete within the host and a single genotype eventually dominates. We assume that these processes of recombination and within-host competition occur on a fast timescale compared to epidemiological events, and are, therefore, instantaneously resolved upon supercolonization to give rise to a host colonized by a single genotype. We denote by *η_i_*_,*j*,*m*→_*_k_* the probability that a colonization by strains *i* and *j* within a host with treatment status *m* gives rise to a majority strain *k*. These probabilities verify $\sum_{k\in R}\eta_{i,j,m\to k}=1$. These probabilities are calculated by first considering whether recombination happens within the host (with probability *ρ*), and second deciding which of the genotypes emerges as the winner, which is affected by the strain's competitive ability *ω_k_*_,*m*_. We define the set ϱ*_i_*_,*j*_ as the set of all genotypes that can be generated by recombination between genotypes *i* and *j*. Then, the probabilities defining supercolonization transitions are

$$\begin{array}{ccc} \eta_{i,j,m\to k}=0 & \text{if}\;k\notin \{i,j\}, & k\notin \varrho_{i,j}\\ \eta_{i,j,m\to k}=\rho \frac{\omega_{k,m}}{\sum_{l\in \varrho_{i,j}}\omega_{l,m}} & \text{if}\;k\notin \{i,j\}, & k\in \varrho_{i,j}\\ \eta_{i,j,m\to k}=\rho \frac{\omega_{k,m}}{\sum_{l\in \varrho_{i,j}}\omega_{l,m}}+(1-\rho )\frac{\omega_{k,m}}{\omega_{i,m}+\omega_{\;j,m}} & \text{if}\;k\in \{i,j\} & \end{array}.$$

The first case corresponds to the situation where genotype *k* is different from both *i* and *j*, and cannot be generated by recombination. The second case corresponds to the situation where genotype *k* is different from both *i* and *j* and must, therefore, be generated by recombination. Recombination happens with probability *ρ*, and will give rise to genotype *k* only if *k* belongs to the set *ϱ_i_*_,*j*_ of genotypes generated by recombination between *i* and *j*. The third case corresponds to the situation where *k* is one of *i* or *j* and can, therefore, be the winning strain in the event of recombination or not. The fractions describe the outcome of within-host competition, whereby a strain *k* present in the host will win the competition between all strains present with a probability equal to its within-host competitive ability *ω_k_*_,*m*_ divided by the summed competitive abilities of all strains present. This model is a simple way to implement differential competitive abilities but does not explicitly describe within-host evolutionary dynamics. In this model, recombination between *i* and *j* is assumed to always generate all intermediate genotypes *ϱ_i_*_,*j*_.

Within-host competitive abilities *ω_k_*_,*m*_ depend on the cost of resistances carried by the bacterial genotype (and the potential interactions between them), and on the effect of treatment on these genotypes (and the potential interaction between drugs). In mathematical terms, the within-host competitive ability for strain *i* under the treatment *m* is *ω_i_*_,*m*_ = (1 − *c_i_*) (1 − ɛ*_i_*_,*m*_). The term 1 − *c_i_* is calculated as the multiplication of all costs of individual resistances and pairwise epistasis between all pairs of resistances. The term 1 − *ɛ_i_*_,*m*_ is calculated as the multiplication of all effects of individual drugs to which genotype *i* is sensitive and interactions for all pairs of drugs.

The instantaneous change in density of a strain *i* under treatment *m* resulting from all the possible events of supercolonization can be written:

$$\begin{array}{rl} & \underline{\underset{\gamma_{i,m}}{\underbrace{\sum_{\;j\in R}\sum_{k\in R}\sum_{n\in T}\sigma \beta_{k,n}X_{k,n}X_{\;j,m}\eta_{\;j,k,m\;\to \;i}}}}\\ & \;-\underset{\pi_{i,m}}{\underbrace{\sum_{\;j\in R}\sum_{k\in R}\sum_{n\in T}\sigma \beta_{k,n}X_{k,n}X_{i,m}\eta_{i,k,m\;\to \;j}}}. \end{array}$$

The first term composed of three sums, denoted by *γ_i_*_,*m*_ (supercolonization gains), is the sum of all possible supercolonizations leading to strain *i* in a host of treatment *m* (all challenger strains *k* × treatments *n* × resident strains *j*). Similarly, the second term, denoted by *π_i_*_,*m*_ (supercolonization losses), is the sum of all possible supercolonizations removing the resident strain *i* under treatment *m* (all possible challenger strains *k* × treatments *n*).

#### 2.1.6. Mathematical form of the model

We model the four following events: colonization of uncolonized (susceptible) hosts, treatment use, clearance of colonized hosts and supercolonization (during which recombination and within-host competition happen on a fast timescale). Hosts can be uncolonized and untreated ($X_{\emptyset ,\emptyset }$), uncolonized and treated ($X_{\emptyset ,m}$), colonized and untreated ($X_{i,\emptyset }$), and, finally, colonized and treated (*X_i_*_,*m*_). The model can be described by the following set of ordinary differential equations:

Uncolonized, untreated individuals:

$$\frac{\text{d}X_{\emptyset ,\emptyset }}{\text{d}t}=\underset{\mathrm{treatment}}{\underbrace{\underset{n\in T^{*}}{\sum }(\nu_{n}X_{\emptyset ,n}-\tau_{n}X_{\emptyset ,\emptyset })}}-\underset{\mathrm{colonization}}{\underbrace{\underset{\;j\in R^{*}}{\sum }\underset{n\in T}{\sum }(\beta_{\;j,n}X_{\;j,n})X_{\emptyset ,\emptyset }}}+\underset{\mathrm{clearance}}{\underbrace{\underset{\;j\in R^{*}}{\sum }u_{j}X_{\;j,\emptyset }}}.$$

Uncolonized, treated with drug *m*:

$$\frac{\text{d}X_{\emptyset ,m}}{\text{d}t}=\underset{\mathrm{treatment}}{\underbrace{\tau_{m}X_{\emptyset ,\emptyset }-\nu_{m}X_{\emptyset ,m}}}-\underset{\mathrm{colonization}}{\underbrace{\underset{\;j\in R^{*}}{\sum }\underset{n\in T}{\sum }(\beta_{\;j,n}X_{\;j,n})X_{\emptyset ,m}}}+\underset{\mathrm{clearance}}{\underbrace{\underset{\;j\in R^{*}}{\sum }(a_{\;j,m}+u_{j})X_{\;j,m}}}.$$

Colonized with strain *i*, untreated:

$$\begin{array}{rl} \frac{\text{d}X_{i,\emptyset }}{\text{d}t} & =\underset{\mathrm{treatment}}{\underbrace{\sum_{n\in T^{*}}(\nu_{n}X_{i,n}-\tau_{n}X_{i,\emptyset })}}+\underset{\mathrm{colonization}}{\underbrace{\sum_{n\in T}(\beta_{i,n}X_{i,n})X_{\emptyset ,\emptyset }}}\\ & \;-\underset{\mathrm{clearance}}{\underbrace{u_{i}X_{i,\emptyset }}}+\underset{\mathrm{supercolonization}}{\underbrace{\gamma_{i,\emptyset }-\pi_{i,\emptyset }}}. \end{array}$$

Colonized with strain *i*, treated with drug *m*:

$$\begin{array}{rl} \frac{\text{d}X_{i,m}}{\text{d}t} & =\underset{\mathrm{treatment}}{\underbrace{\tau_{m}X_{i,\emptyset }-\nu_{m}X_{i,m}}}+\underset{\mathrm{colonization}}{\underbrace{\sum_{n\in T}(\beta_{i,n}X_{i,n})X_{\emptyset ,m}}}\\ & \;-\underset{\mathrm{clearance}}{\underbrace{(a_{i,m}+u_{i})X_{i,m}}}+\underset{\mathrm{supercolonization}}{\underbrace{\gamma_{i,m}-\pi_{i,m}}}. \end{array}$$

### 2.2. Linear discriminant analysis of the simulation results

We investigated with simulations the model's behaviour as a function of its parameters. We ran 400 000 simulations of the model with randomly chosen sets of parameters and recorded the equilibrium values. Parameters were randomly chosen in plausible distributions (table 1). The rates of antibiotic clearance *a_i_*_,*m*_ were set to 20 per month when clearance is successful (treatment *m* clears strain *i*) and 0 otherwise.

To analyse the results of simulations, we used linear discriminant analysis (LDA; as encoded in the MASS package [30] in R [31]). LDA reduces the high-dimensional parameter space into a low dimensional space that best separates the outcome of the model in terms of a qualitative variable of interest (see [32] for an application of LDA to a similar topic). The qualitative variable that we used was the list of all strains present at a frequency greater than 1% in the system after 300 months, a time when the equilibrium of the system is reached (electronic supplementary material, figure S2). The basis of the reduced space (here, we chose to reduce space to two dimensions) is composed of linear combinations of these parameters. The impact of each parameter on the outcome of the model can thus be described by a vector of the contribution of this parameter on each dimension.

To check the robustness of the results, we bootstrapped the 400 000 simulations, and visually confirmed that the LDA gave similar results (electronic supplementary material, figure S3).

### 2.3. Basic reproductive number and invasion fitness

In addition to simulations, to gain analytical understanding of the model, we computed the basic reproductive number *R*_0,*i*_ of a strain *i*, as well as the invasion fitness *r_i_*_,*j*_ of strain *j* invading a host population already colonized by the wild-type strain *i* equilibrium. The basic reproductive number *R*_0,*i*_ quantifies the number of newly colonized individuals per colonized individual in an otherwise fully susceptible host population. It is a relevant quantity to evaluate the spread of a transmissible disease in a fully susceptible (uncolonized) population. When *R*_0,*i*_ > 1, the strain *i* spreads in the population. However, *R*_0_ might not be a very useful quantity to evaluate the spread of a new genotype (for example, a resistant strain) in a population where another genotype (for example, a sensitive strain) is already present [33]. We will see below that *R*_0,*i*_ is a relevant quantity to determine which strain becomes dominant only in an approximation of the above model. The invasion fitness *r_i_*_,*j*_ is the growth rate of rare strain *j* invading a host population where another strain *i* is already present and at equilibrium [34]. When positive, the strain *i* will invade and reach a positive equilibrium. Furthermore, if *r_i_*_,*j*_ > 0 and *r_j_*_,*i*_ > 0, both strains *i* and *j* can invade the population of the other when rare, meaning that both strains can coexist [18,35].

All analyses and simulations, with the exception of the LDA, were done with Wolfram Research, Inc., Mathematica, v. 11.3, Champaign, IL (2018).

## 3. Results

Our aim is to describe the bacterial strain composition at the equilibrium as a function of the model parameters. To this end, we simulated the model under randomly chosen sets of parameter values, and complemented these simulations with an analysis of the strains' basic reproductive number and invasion fitness.

In the first part of the paper, we consider the dynamics of a bacterial species in a single, well-mixed population of hosts. Three antibiotic treatments are applied to the host population: a treatment by antibiotic 1 denoted by *T*_1_, a treatment by a distinct antibiotic 2 denoted by *T*_2_ and combination therapy (denoted by *T*_1,2_). Thus, the bacterial population is in contact with the two drugs. The bacterial population is composed of four strains: the fully sensitive strain *S*, the strain *R*_1_ resistant to the drug 1 only, the resistant *R*_2_ resistant to the drug 2 only and the double resistant strain *R*_1,2_ resistant to both drugs. In the second part, we relax the assumption of a single well-mixed population and consider the evolution of resistance in two classes of hosts taking antibiotics at different rates.

### 3.1. Factors favouring the evolution of resistant and multi-resistant strains

#### 3.1.1. An advantage for multi-resistance over single resistances

**Figure 1.**
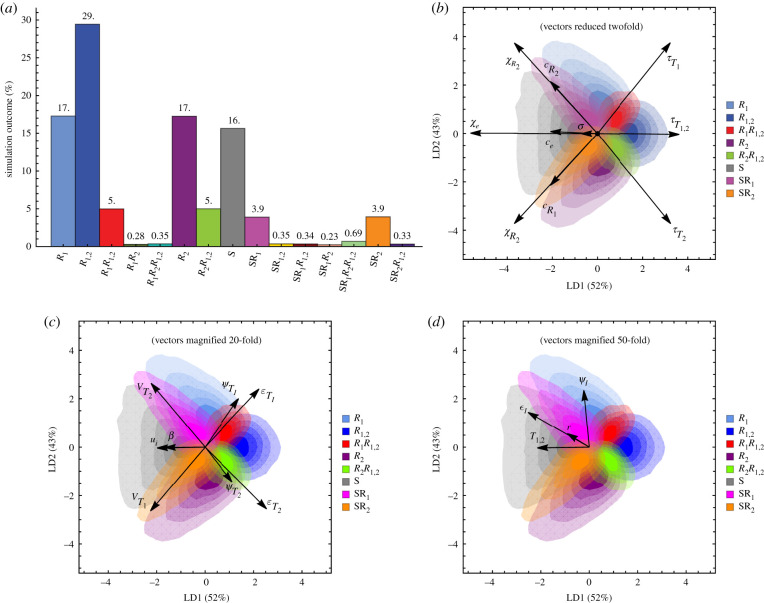
Results of the linear discriminant analysis. (*a*) The frequency (in %) of occurrence of each combination of strains among the 400 000 simulations. We considered that a strain was present when its prevalence at 300 months (a time when equilibrium was reached) was greater than 1%. (*b–d*) The output of the LDA for the most frequent outcomes. For readability, parameters are presented on three different panels (*b*-*d*) from greatest to lowest impact on the outcome. The opacity levels of the different colours represent the density of the cloud points associated with each strain combination. The arrows show the contributions of parameters to each of the directions. In (*b*), pairwise transmission cost epistasis and within-host cost epistasis are denoted by *χ_e_* and *c_e_*, respectively. In (*d*), and within-host drug interactions parameters are denoted by *ψ_I_* and *ɛ_I_*, respectively. The results were generated with 400 000 randomly chosen sets of parameters (table 1). The effects of transmission and within-host drug interaction *ɛ_I_* and *ψ_I_*, and of recombination *r* were not consistent across bootstrapped simulations (panel *d*), in contrast with all other parameters (electronic supplementary material, figure S3).

In the single well-mixed population model, the evolution of double resistance was the most common outcome (figure 1*a*). For randomly chosen parameter values (table 1), at equilibrium, 29% of the 400 000 simulations resulted in the double resistant strain dominating. Furthermore, coexistence between sensitive and multiple resistant bacterial strains was rarely maintained. A single strain was maintained in 80% of the simulations. In rarer cases, two strains coexisted at equilibrium (representing 18% of parameters), while coexistence between more than two strains represented 1.9% of parameters. This property of models of drug resistance evolution has been identified before in simpler models [36,37], and, for the most part, holds true in this more complicated model that includes an explicit description of treatment dynamics and supercolonization. Intuitively, several strains cannot easily coexist because sensitive and resistant strains compete to colonize the same hosts.

These results can be understood by analysing a simplified model where treatment is *implicit* and there is no supercolonization. By *implicit*, we mean that treatment instantaneously clears sensitive strains (thus decolonizing the host) and has no effect at all on resistant strains. In such a simplified model, no coexistence is maintained at equilibrium and the winning strain is the one with the highest basic reproductive number or *R*_0,*i*_ (electronic supplementary material, sup. text). In this simplified model, we find that the basic reproductive number of strain *i* is

$$R_{0,i}=\frac{\beta_{i,\emptyset }}{u_{i}+\sum_{m\in T^{*}}\delta_{i,m}\tau_{m}},$$

where *δ_i_*_,*m*_ is an indicator variable defining the sensitivity status of strain *i*, equal to 1 when strain *i* is sensitive, and 0 when strain *i* is resistant to treatment *m*. The strain striking the best balance between the cost of resistance (that may both reduce the transmission rate $\beta_{i,\emptyset }$ and increase the clearance rate *u_i_*) and the benefits of resisting treatments will be the eventual winner.

The analysis of basic reproductive numbers captures reasonably well the domain of dominance of a strain. This was verified in additional simulations with chosen parameters (electronic supplementary material, figure S1). This was true even in the full simulated model, which differs by the consideration of explicit treatment, supercolonization and recombination (electronic supplementary material, figure S1B,C).

While the evolution of double resistance was the most frequent outcome, it was very rare that the two single resistant strains were present in the population at equilibrium. The examination of the basic reproductive numbers suggests that when both single resistances fare better than the sensitive strain, the double resistant strain should eventually dominate (electronic supplementary material, sup. text). Thus, interestingly, in a commensal bacterial population evolving in a host population subjected to multiple treatments, the evolution of double resistance may be a common outcome even in the absence of combination therapy. This confirms one of the hypotheses for the evolution of MDR in commensal bacteria (hypothesis (ii) in the introduction).

#### 3.1.2. Linear discriminant analysis of the single population model

The LDA reveals which parameters are important to the evolution of resistant and multi-resistant strains (figure 1*b*). The LDA identifies domains where a certain dynamical outcome predominates in the parameter space. In our analysis, the horizontal axis separated sensitivity from double resistance. The vertical axis separated resistance to drug 1 from resistance to drug 2. In addition, each parameter can be represented as a vector in the reduced space. The norm of the vectors quantifies the importance of the corresponding parameter for determining the final outcome, given the range of parameters sampled.

In line with the results derived from the basic reproductive number, the best predictors of the success of a resistant strain were a high rate of the corresponding treatment (high *τ_m_*), a long duration of this treatment (small *ν_m_*) and a low cost of resistance in terms of transmission and within-host competition (figure 1*b*). Combination therapy (corresponding to the rate of treatment $\tau_{T_{1,2}}$), in particular, favoured the evolution of the double resistant strain as expected.

The costs of resistance had a strong influence on the outcome (i.e. had the largest norms) (figure 1*b*). Among these costs, for our choice of parameters, the cost on transmission *χ_i_* hindered resistant strains more than the cost on within-host competitive ability *c_i_*. This is because the cost on within-host competitive ability is only expressed upon supercolonization, which in our model occurred less frequently than transmission to an uncolonized host (this is modulated by the susceptibility to supercolonization *σ*, table 1). Furthermore, the double resistant strain was favoured when its cost was lower than expected from the two single resistances combined. This is evident on figure 1*b* from the vectors representing cost epistasis, be it on transmission (parameter *χ_e_*) or on within-host competitive ability (parameter *c_e_*). Negative cost epistasis is indeed one of the hypotheses for the evolution of MDR (hypothesis (i) in the introduction).

The LDA also shows that low transmission, low supercolonization and long carriage duration (low *u_i_*) all favoured MDR (figure 1*b*,*c*). To understand why, we computed the invasion fitness of different strains. The invasion fitness of a mutant is its initial growth rate when invading a resident strain at equilibrium. When it is positive, the mutant will successfully establish in the population. The analytical expressions for invasion fitness of a strain given its resistance profile inform on the factors that favour sensitivity, single resistance of multiple resistance in the model (including explicit treatment dynamics and supercolonization, in contrast with the analysis based on basic reproductive numbers). Our main analytical result is the approximation of the invasion fitness under three assumptions: (i) the treatment rates are smaller than epidemiological rates (transmission, clearance), (ii) the probability of supercolonization *σ* is small and (iii) recombination is ignored.

Under these assumptions (details in electronic supplementary material, Mathematica notebook), the invasion fitness of a challenger strain *j* invading a resident strain *i* denoted *r_i_*_,*j*_ is, to the first order:

$$\begin{array}{rl} r_{i,j}= & \underset{\begin{array}{c} \mathrm{colonization}\;\;\mathrm{of}\;\\ \mathrm{untreated}\;\;\mathrm{hosts} \end{array}}{\underbrace{\beta_{\;j,\emptyset }{\tilde{X}}_{\emptyset ,\emptyset }}}-\underset{\begin{array}{c} \mathrm{natural}\;\\ \mathrm{clearance} \end{array}}{\underbrace{u_{j}}}+\underset{\begin{array}{c} \mathrm{colonization}\;\mathrm{of}\;\\ \mathrm{treated}\;\;\mathrm{hosts} \end{array}}{\underbrace{\sum_{m\in T^{*}}\Delta_{\;j,m}\;\beta_{\;j,\emptyset }\;{\tilde{X}}_{\emptyset ,m}}}\\ & -\underset{\begin{array}{c} \mathrm{antibiotic}\;\\ \mathrm{clearance} \end{array}}{\underbrace{\sum_{m\in T^{*}}(1-\Delta_{\;j,m})\tau_{m}}}+\underset{\mathrm{supercolonization}\;\mathrm{balance}}{\underbrace{\sigma \;{\tilde{X}}_{i,\emptyset }(\beta_{\;j,\emptyset }\eta_{i,j,\emptyset \to j}-\beta_{i,\emptyset }\eta_{\;j,i,\emptyset \to i})}}. \end{array}$$

where ${\tilde{X}}_{i,m}$ denotes the density of hosts colonized by the strain *i* under treatment *m*, when the resident strain *i* is at equilibrium. In this expression, the first two terms correspond to the growth rate of strain *j* in the population at equilibrium, in the absence of treatment and supercolonization. This growth rate results from a balance between colonization of untreated hosts and natural clearance. The third term represents colonization of treated uncolonized hosts. It is summed over all possible antibiotic treatments and modulated by $\Delta_{\;j,m}=(\beta_{\;j,m}{\tilde{X}}_{\emptyset ,\emptyset }+\nu_{m})/(\beta_{\;j,\emptyset }{\tilde{X}}_{\emptyset ,\emptyset }+\nu_{m}+a_{\;j,m})$. The fourth term represents antibiotic clearance, again summed over all antibiotics, and again modulated by Δ*_j_*_,*m*_. The quantity Δ*_j_*_,*m*_ is between zero and one in the biologically plausible case where the transmission rates are smaller in treated individuals ($\beta_{\;j,m}\leq \beta_{\;j,\emptyset }\;\forall \;m$). For a perfectly sensitive strain *a_j_*_,*m*_ → ∞, Δ*_j_*_,*m*_ = 0: this strain cannot transmit to treated individuals and is completely cleared by antibiotics. For a perfectly resistant strain (*a_j_*_,*m*_ = 0, $\beta_{\;j,m}=\beta_{\;j,\emptyset }\;\forall \;m\in \;T$), Δ*_j_*_,*m*_ = 1: the strain can fully transmit to treated individuals and is not cleared by antibiotics. The last term in the equation represents the balance resulting from supercolonization events, where $\eta_{i,j,\emptyset \to j}$ is the probability that supercolonization by a strain *j* of an untreated (the index ∅) host colonized by a strain *i*, leads to a host colonized by strain *j* following within-host competition. Similarly, $\eta_{\;j,i,\emptyset \to i}$ is the probability that supercolonization by strain *i* of an untreated host colonized by a strain *j*, leads to a host colonized by strain *i* following within-host competition. Under the assumptions of small treatment rates and small probability of supercolonization, the supercolonization of treated hosts that potentially favours resistant strains is a negligible phenomenon. We ignored recombination to obtain the equation. Recombination would potentially create other strains than the resident *i* and the challenger *j*, which would greatly complicate the analysis. Later on, we use simulations to investigate the impact of recombination of evolutionary dynamics of MDR.

To further facilitate the interpretation of the expression for the invasion fitness, we express it for the strain *R*_1,2_ perfectly resistant to the two antibiotics (no effect of treatments on clearance or on transmission, $a_{R_{1,2},m}=0\;$ and $\beta_{R_{1,2},m}=\beta_{R_{1,2},\emptyset },\forall \;m\in T$), invading a perfectly sensitive strain *S* at equilibrium (*a_S_*_,*m*_ → ∞, ∀ m∈T). Under these assumptions, invasion fitness simplifies to:

rS,R1,2=βR1,2,∅X~∅,∅⏟colonizationofuntreatedhosts−uR1,2⏟naturalclearance+βR1,2,∅(X~∅,T1+X~∅,T2+X~∅,T1,2)⏟colonization of treated hosts+σ X~S,∅(βR1,2,∅ηS,R1,2,∅→R1,2−βS,∅ηR1,2,S,∅→S)⏟supercolonizationbalance.

where $r_{S,R_{1,2}}$ is the invasion fitness of the double resistant strain *R*_1,2_ invading the sensitive strain *S* at equilibrium. The double resistant strain invades the sensitive strain at equilibrium by multiplying in both untreated and treated individuals. The double resistant strain is not hindered by treatment. However, it may suffer a cost in the form of a reduced transmission rate compared to the sensitive strain ($\beta_{R_{1,2},\emptyset }<\beta_{S,\emptyset }$). It also suffers a cost in supercolonization, as its within-host competitive ability is reduced in untreated hosts: thus, the supercolonization balance is negative for the double resistant strain.

Analogously, the sensitive strain invading the doubly resistant strain has invasion fitness:

$$\begin{array}{rl} r_{R_{1,2},S}= & \underset{\begin{array}{c} \mathrm{colonization}\;\;\mathrm{of}\\ \mathrm{untreated}\;\;\mathrm{hosts} \end{array}}{\underbrace{\beta_{S,\emptyset }{\tilde{X}}_{\emptyset ,\emptyset }}}-\underset{\begin{array}{c} \mathrm{natural}\;\\ \mathrm{clearance} \end{array}}{\underbrace{u_{S}}}-\underset{\begin{array}{c} \mathrm{antibiotic}\;\\ \mathrm{clearance} \end{array}}{\underbrace{\left(\tau_{T_{1}}+\tau_{T_{2}}+\tau_{T_{1,2}}\right)}}\\ & +\underset{\mathrm{supercolonization}\;\;\mathrm{balance}}{\underbrace{\sigma \;{\tilde{X}}_{R_{1,2},\emptyset }(\beta_{S,\emptyset }\eta_{R_{1,2},S,\emptyset \to S}-\beta_{R_{1,2},\emptyset }\eta_{S,R_{1,2},\emptyset \to R_{1,2}})}}. \end{array}$$

where conversely, $r_{R_{1,2},S}$ is the invasion fitness of the sensitive strain *S* invading the double resistant strain *R*_1,2_ at equilibrium. The sensitive strain grows only in untreated individuals and is cleared by treatment, but does not suffer a cost.

These analytical expressions for invasion fitness delineated when a strain was able to invade another (electronic supplementary material, figure S1, for the example of coexistence of a sensitive and double resistant strain). It also explained the rare areas of coexistence of two strains observed in the simulations (figure 1). Indeed, when two strains can reciprocally invade the other, that is *r_i_*_,*j*_ > 0 and *r_j_*_,*i*_ > 0, coexistence between these two strains is possible. Sensitive and resistant strains use different strategies. The sensitive strain multiplies efficiently in the untreated hosts, while the resistant strains multiply less efficiently but both in untreated and treated individuals. In other words, the treatment structure means the environment of the pathogen is multidimensional and coexistence of sensitive and resistant strains can in theory occur [33]. In practice, coexistence was a rare outcome in our simulations.

**Figure 2.**
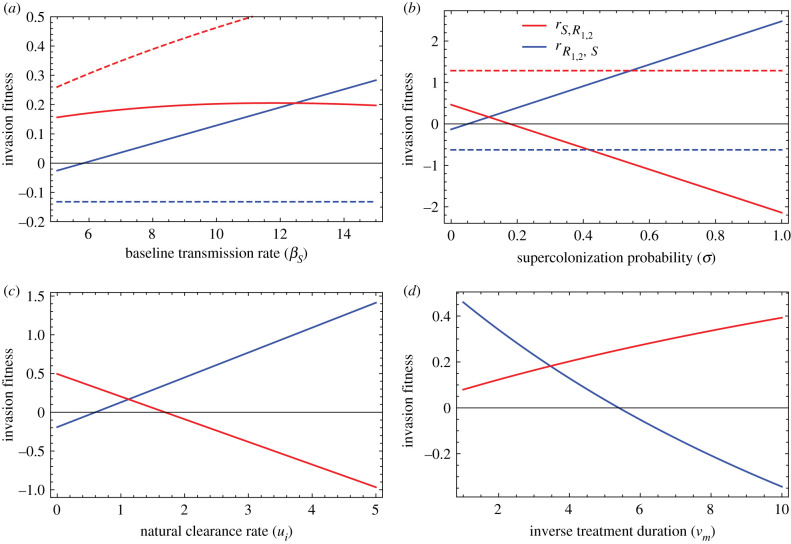
Invasion fitnesses of the sensitive strain *S* (blue lines) and the double resistant strain *R*_1,2_ (red lines) invading the other strain at equilibrium. A positive value means that the invading strain can establish. In (*a*), the invasion fitness is shown with supercolonization (plain lines, *σ* = 0.1) and without supercolonization (dashed lines, *σ* = 0). In (*b*), the invasion fitness is shown with within-host and transmission costs of resistance (plain lines, $\chi_{R_{1}}=c_{R_{1}}=\chi_{R_{2}}=c_{R_{2}}=0.2$) and without these costs (dashed lines, $\chi_{R_{1}}=c_{R_{1}}=\chi_{R_{2}}=c_{R_{2}}=0$). In (*d*), when varying the inverse treatment duration (*ν_m_*), the expected fraction of treated individuals *τ_m_*/*ν_m_* is kept constant and equal to 0.05. Unless specified otherwise, parameter values in all panels are: $\chi_{R_{1}}=c_{R_{1}}=\chi_{R_{2}}=c_{R_{2}}=0.2$. Epistasis effects are set to 0. Effects of treatment on within-host competitive ability and transmission *ɛ_i_*_,*m*_ = *ψ_i_*_,*m*_ = 0.75 when the strain *i* is sensitive to treatment *m*, and drug interactions effects are set to 0. *β* = 10, $u_{i}=1\;\forall \;i\;$, *a_i_*_,*m*_ = 20 when strain *i* is sensitive to treatment *m* and 0 otherwise, *σ* = 0.1, *τ_m_* = 0.2 and $\nu_{m}=4\;\forall \;m\in \;T$.

The effects of transmission, supercolonization and carriage duration can be understood from the analysis of invasion fitness:

— The effects of transmission on resistance are complex. The main effect of increased transmission is to favour the invasion of the sensitive strain (blue line on figure 2*a*) because of the advantage it enjoys in supercolonization. Indeed, the sensitive strain is fitter than the resistant and can displace it from a colonized host. Without supercolonization, the sensitive strain is unaffected by transmission (figure 2*a*, blue dashed line). The increased transmission also favours (but to a far lesser extent) the resistant strain, because it transmits more readily to treated hosts [18]. Without supercolonization, this is the dominant effect and increased transmission favours the resistant strain (figure 2*a* red dashed line).
— Supercolonization favours the sensitive strain (figure 2*b*). Competition through supercolonization favours the sensitive strain because it transmits more and it is fitter than the resistant strains within untreated host. This benefit of supercolonization for the sensitive strain depends on the costs of resistance on transmission *χ_i_* and within-host competitive ability *c_i_*: in additional simulations where we set these costs equal to zero, the benefit of supercolonization disappears (figure 2*b* dashed lines).
— Longer carriage duration (smaller natural clearance rate) favours the resistant strain (figure 2*c*) [38].

The slope of the relationship between invasion fitness and a parameter reflects the strength of the effect of this parameter. These slopes (figure 2) are consistent with the LDA: supercolonization has a large effect compared to both transmission and carriage duration.

Lastly, and interestingly, a strategy consisting of more frequent treatments (high treatment rates *τ_m_*) with shorter duration (high rate of treatment cessation *ν_m_*) while keeping the fraction of treated individuals constant advantages the double resistant strain (figure 2*d*). This new result is an extension to multiple treatments of a previous result according to which more frequent, shorter-duration treatment favours resistant over sensitive strains. More frequent, shorter treatments disadvantages sensitive strains because more hosts are cleared of the sensitive strains [18].

## 3.2. Evolution of MDR in a structured host population

The structure of the host population may affect the evolution of MDR. Hosts of different ages, living in different locations, in the community or in the hospital, represent a heterogeneous environment in which multiple bacterial strains can multiply. Host structure favours coexistence of multiple strains when the different classes are treated at very different rates and when they are strongly isolated [18]. To study how structure in the host population influences the evolution of MDR, we developed a two-class extension of our model. We used 400 000 additional simulations of this extended model with randomly chosen parameter sets to examine how MDR evolves in these two host classes. The rate of treatment start, the rate of treatment stop and the duration of carriage vary across the two classes (table 2).

**Table 2.** Sampling distributions of the parameters in the two-classes model (other parameters as in the well-mixed single population model). We explore a wide range of proportion of inter-class transmission. For example, transmission between individuals from different regions or countries is likely to be small. The transmission rate between individuals of different age classes is around 10–50% that within age classes [39]. Sampling ranges for other parameters are justified as for the single population model.

parameter	meaning	sampling range	sampling method
*α_x_*_,*y*_, *x* ≠ *y*	proportion of inter-class transmission from class *y* to *x*	[10^−3^, 1]	logarithmic
*α_y_*_,*y*_	proportion of intra-class transmission in class *y*	$1-\underset{x}{\sum }\alpha_{x,y}$	—
$\tau_{T_{1},A}$	rate of treatment *T*_1_ in class A	[0, 0.2]	uniform
$\tau_{T_{1},B}$	rate of treatment *T*_1_ in class B	[2 × 10^−4^, 2 × 10^−2^]	logarithmic
$\tau_{T_{2},A}$	rate of treatment *T*_2_ in class A	[2 × 10^−4^, 2 × 10^−2^]	logarithmic
$\tau_{T_{2},B}$	rate of treatment *T*_2_ in class B	[0, 0.2]	uniform
$\tau_{T_{1,2},x}$	rate of treatment *T*_1,2_ in class *x*	[2 × 10^−4^, 0.2]	logarithmic
*ν_m_*_,*x*_	rate of end of treatment *m* in class *x*	[1, 6]	uniform
*u_i_*_,*x*_	natural clearance rate of strain *i* in class *x*	[0.1, 3]	uniform (same for all strains)

The set of all classes is denoted by C. The pathogen moves across host classes by inter-class transmission, which we assume is typically smaller or equal to intra-class transmission. To control these rates of transmission, we denote by *β_i_*_,*m*,*y*→_*_x_* = *α_x_*_,*y*_*β_i_*_,*m*_ the transmission rate of strain *i* colonizing a host of treatment *m* from class *y* to a host of class *x*. The parameter *α_x_*_,*y*_ is the fraction of all transmission from hosts in class *y* that affect hosts in class *x*. Thus, ∀ y∈ C, $\sum_{x\in C}\alpha_{x,y}=1$. Two classes *x* and *y* are fully isolated if *α_x_*_,*y*_ = *α_y_*_,*x*_ = 0. The population is homogeneously mixed if $\forall \;(x,y)\in \;C^{2}$, *α_x_*_,*y*_ = 1/*N_C_* where *N_C_* is the number of classes. Here, we consider two classes A and B.

Our simulations show that aligned use of the two antibiotics across classes favours MDR. When the host population is structured, the rates of treatment by antibiotic 1 and 2 in the different classes are now vectors $\tau_{T_{1}}$ and $\tau_{T_{2}}$ whose elements are the values $\tau_{T1,i}$ and $\tau_{T2,i}$ in each class *i* of host. These vectors are perfectly aligned when $\tau_{T_{1}}$ is proportional to $\tau_{T_{2}}$, in which case the classes differ only by the intensity of prescription of all antibiotics. This scenario promotes the evolution of MDR if the classes are sufficiently isolated, because MDR evolves in the classes with high use of all antibiotics [17]. By contrast, when the two vectors are orthogonal ($\tau_{T_{1}}\cdot \tau_{T_{2}}=0$), at most one antibiotic is used in each class. This scenario should instead promote the evolution of single resistances in each class, if the classes are sufficiently isolated. To verify this intuition, we computed the cosine of the angle between the antibiotic use vector as $\tau_{T_{1}}\cdot \tau_{T_{2}}/(∥\tau_{T_{1}}∥∥\tau_{T_{2}}∥)$. This cosine varies from 0 (aligned uses) to 1 (orthogonal uses).

The effect of the alignment of antibiotic uses across classes on MDR was evident on the LDA of the 400 000 simulations of the two-classes model (electronic supplementary material, figure S4). Furthermore, the LDA also shows, just as in the single population model, that the evolution of double resistance in the two-classes model is favoured by high rates of treatments, combination therapy, low costs of resistance, negative cost epistasis, low supercolonization.

**Figure 3.**
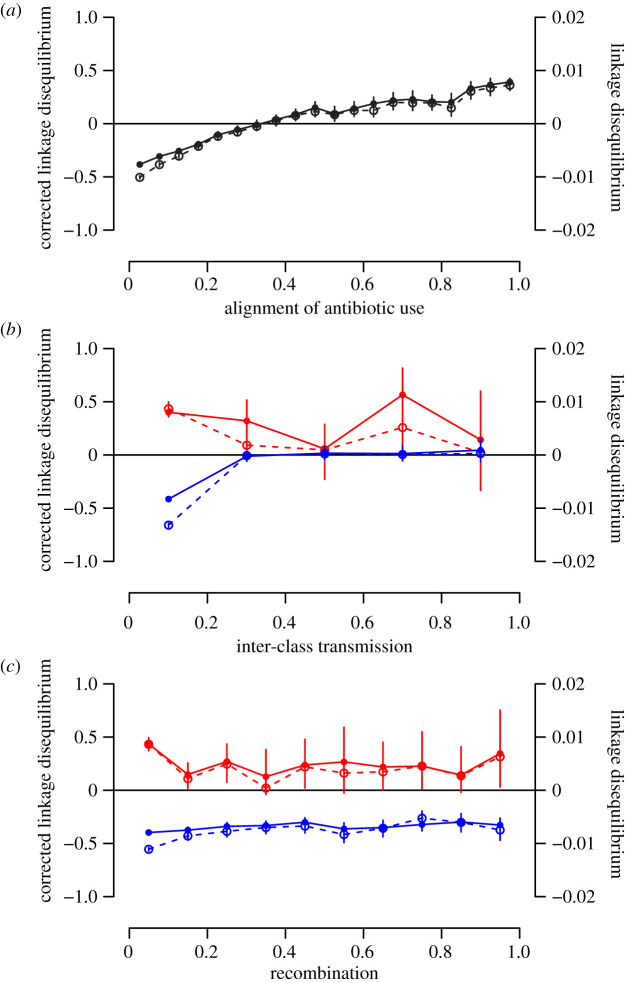
Linkage disequilibrium as a function of alignment of antibiotic use (*a*), inter-class transmission (*b*) and recombination (*c*) in a two-classes population model. Both the linkage disequilibrium (dashed line, open circles) and corrected linkage disequilibrium (filled line, filled circles) are shown. (*a*) Linkage disequilibrium as a function of the cosine of the angle between use of antibiotic 1 and use of antibiotic 2. (*b*) Linkage disequilibrium as a function of inter-class transmission *α_x_*_,*y*_. (*c*) Linkage disequilibrium as a function of recombination *r*. In (*b*,*c*), we focused on subsets of the simulations with highly aligned antibiotic uses (cosine of the angle greater than 0.95, red) and orthogonal uses (cosine of the angle smaller than 0.05, blue). In all panels, we used the simulations of the extended two-classes model with randomly chosen parameter sets. We show the mean linkage disequilibrium in bins. The vertical lines represent the bootstrap confidence intervals. The corrected linkage disequilibrium is shown for the subset of 41 696 simulations where polymorphism is not lost.

To further quantify MDR, we computed the linkage disequilibrium between the two resistance loci. Calling the frequencies of each bacterial genotype *f_S_*, $f_{R_{1}}$, $f_{R_{2}}$, $f_{R_{1,2}}$, linkage disequilibrium is defined as $LD=f_{R_{1,2}}\;f_{s}-f_{R_{1}}f_{R_{2}}$. LD varies between −0.25 (resistances are found in single resistant genotypes only) and +0.25 (resistances are found in double resistant genotypes only). LD declines with the level of polymorphism, and is 0 when there is no polymorphism at either locus. We also define the *corrected* linkage disequilibrium as $LD^{\prime }=LD/\sqrt{\;p_{1}(1-p_{1})p_{2}(1-p_{2})}$ where $p_{1}=f_{R_{1}}+f_{R_{1,2}}$, and $p_{2}=f_{R_{2}}+f_{R_{1,2}}$. The corrected LD varies from −1 (resistances are found in single resistant genotypes only) to +1 (resistances are found in double resistant genotypes only) and is unaffected by the level of polymorphism. We found that the mean linkage disequilibrium increased with the alignment of use of antibiotics 1 and 2 (figure 3*a*).

By focusing on the subset of simulations with very aligned uses (cosine of the angle greater than 0.95) and those with very orthogonal uses (cosine of the angle less than 0.05), we found that the emergence of positive or negative linkage disequilibrium depends on the classes being isolated one from another (inter-class transmission has to be much smaller than intra-class transmission) (figure 3*b*). However, the rate of recombination had little effect on linkage disequilibrium, both for aligned and orthogonal antibiotic uses (figure 3*c*). The build-up of positive or negative LD depends on different genotypes being locally favoured in one or the other class. For aligned uses, *R*_1,2_ is favoured in one class and *S* is favoured in the other. For orthogonal use, *R*_1_ is favoured in one class and *R*_2_ is favoured in the other. In both cases, this results in LD emerging at the whole population level. Large inter-class transmission erodes polymorphism, which reduces LD. In the cases when polymorphism is maintained at high inter-class transmission, it is maintained by mechanisms other than population heterogeneity among classes (i.e. treated individuals providing a niche for the resistant strain and/or supercolonization providing a frequency-dependent advantage to the sensitive strain), which do not generate LD. This results in the decline of corrected LD with inter-class transmission. Because LD is generated by heterogeneous selection across classes and limited mixing, there is little effect of recombination on LD: within each class the genetic diversity is small, and therefore recombination does not have an impact.

## 4. Discussion

We examined the factors favouring the evolution of MDR in commensal bacteria. We used an epidemiological-evolutionary model describing transmission, natural clearance, supercolonization, within-host competition and recombination. We investigated how bystander selection by antibiotic treatments selects for single or multiple resistance in commensal bacteria.

In the introduction, we recalled three non-mutually exclusive hypotheses for the proliferation of MDR strains: (i) negative cost epistasis, whereby the overall cost of MDR may be lower than predicted from the costs of individual resistances; (ii) the use of multiple antibiotics in the host population; and (iii) the structure in the host population. We confirmed the three hypotheses and showed that:

— With the range of parameters chosen in our simulations, negative cost epistasis was the most potent factor selecting for MDR (figure 1*b*, parameters *χ_e_* and *c_e_*);
— Perhaps our most important result is that the application of several single drug treatments in the host population favoured MDR as much as combination therapy. Combination therapy of course favours MDR, but is comparatively rarer (figure 1*b*, table 1). In other words, MDR may evolve even when drugs are never taken in combination in individual hosts (in agreement with previous results from a simpler model [17]).
— In a structured host population, alignment in antibiotic use across classes favours MDR (figure 3*a*, electronic supplementary material, figure S3C).

Negative cost epistasis means that the overall cost of two resistances combined on a MDR genotype is lower than expected. This is most often called ‘positive epistasis’, as this corresponds to fitter than expected MDR genotypes. Positive epistasis between resistance mutations is common [40]. Several studies evaluated the costs of resistance and epistasis by measuring the fitness of sensitive, resistant and MDR strains in the absence of antibiotic *in vitro* [41,42]. For example, Trindade *et al*. [41] generated *E. coli* mutants resistant to the antibiotics nalidixic acid, rifampicin and streptomycin. Seventy-three percent of double mutants with significant epistasis had *positive* epistasis. Thus, MDR genotypes with these low-cost pairs of resistance mutations could readily evolve if the cost measured on the growth rate *in vitro* is a good proxy for the within-host and epidemiological costs of our model. Consistent with this idea, the pairs of resistance mutations that had the lowest *in vitro* costs were also those found most frequently in clinical isolates of *M. tuberculosis* [43].

We emphasize several assumptions of our model. First of all, we assumed that antibiotic treatment was given independently of the colonization status of the host (bystander exposure). We indeed consider commensal species that are most of the time carried asymptomatically and rarely cause infections. Treatment is given for another infection that is not modelled. However, for some drug-species combinations, the proportion of antibiotic exposure that is targeted to the species may be significant. For example, close to 20% of *E. coli*'s exposure to quinolones is targeted rather than bystander exposure [16]. Indeed, urinary tract infections are commonly caused by *E. coli* and treated with quinolones. When antibiotic use is targeted, the resistance of the focal species to the first-line drug may result in treatment failure and use of a second drug. This sequential pattern of antibiotic use, not considered in the present model, could further promote MDR because single resistant strains would be more frequently exposed to a drug they are sensitive to. Second, we assumed for simplicity that only one strain could colonize a host at a time. Within-host competition between strains was assumed to be strong and to rapidly result in a single strain taking over the host. However, in some species with high prevalence like *E. coli*, multiple strains are frequently present, and can coexist over long time periods in a host [25,44]. In these species, a costly resistant strain in competition with a sensitive strain within a host could decline slowly or persist at low frequency. Less intense within-host competition could weaken the effect of within-host parameters on MDR evolution. It would also affect how transmission and supercolonization influence the evolution of resistance, as discussed below.

### 4.1. The role of transmission and supercolonization on MDR

Interestingly, higher transmission favoured the sensitive strain (figure 1*d*). This opposes the frequent assumption that high transmission (e.g. poor hygiene) favours the proliferation of antibiotic resistance. A small number of empirical studies suggest that higher transmission favours resistance. Antibiotic resistance of several bacterial species was found to be associated with poorer infrastructure and poorer governance across 103 countries [45]. One interpretation of this correlation is that the dissemination of resistance is favoured by poor sanitation. More convincingly, the individual carriage of extended-spectrum beta-lactamase-producing *E. coli* (an enzyme conferring resistance to beta-lactams antibiotics) was strongly associated with an indicator of poor hygiene in a large cohort [46]. However, it is not at all obvious in theory why transmission would favour resistance. Larger transmission implies that *both* the resistant and the sensitive strain transmit more. Why would this preferentially favour the resistant strain? In a previous study, it was shown that transmission favours resistance because resistant strains, unlike sensitive strains, rely on transmitting to treated individuals for multiplication [18]. In the model analysed here, this first effect is present (figure 2*a*) but is overwhelmed by a second effect: the *sensitive* strain is favoured by transmission and supercolonization of already occupied hosts, because it enjoys a fitness advantage in competition with the resistant strain within the host (as in the model presented in [47]). In our simulations, that second effect is stronger than the first, and higher transmission, therefore, favours the sensitive strain. It is possible that in the contexts of the empirical studies mentioned above, supercolonization and within-host competition are less common than in the range of parameters explored in the simulations. The higher transmission would thus favour resistant strains as they are able to colonize empty treated hosts.

**Figure 4.**
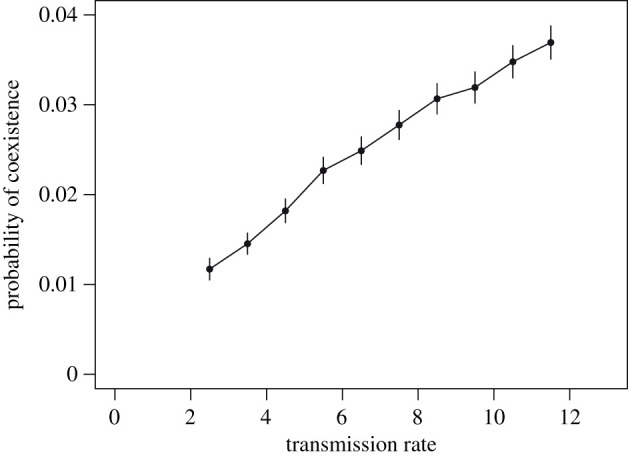
The probability of coexistence of multiple sensitive and resistant bacterial strains increases with the transmission rate. The probability that both resistances coexist at intermediate frequency (in [0.01, 0.99]) across the 400 000 single population simulations is shown as a function of the transmission rate category (10 bins), with binomial confidence intervals.

Because of the two effects, larger transmission favours the invasion of a sensitive strain by a resistant, and reciprocally (figure 2*a*, both invasion fitnesses increase with transmission). Thus, large transmission should also promote the coexistence of multiple strains. This prediction was verified in the simulations: the probability that both resistances coexist with sensitivity (frequency of both resistances in [0.01, 0.99]) increases with transmission (figure 4).

The effects of transmission and supercolonization on the dynamics of resistance and sensitivity are complex and would be interesting to elucidate further. For example, introducing the possibility of long-term coexistence between multiple strains within hosts could affect our results. In a theoretical study, it was shown that co-colonization with resistant and sensitive strains favours the resistant strain because the elimination of the sensitive strain upon antibiotic treatment releases the resistant strain [47]. The transmission rate, therefore, impacts the competition between sensitive and resistant strains in three ways: through the increased transmission of the resistant strain in treated hosts, through increased supercolonization (which favours the sensitive strain if it has an advantage in supercolonization) and through increased co-colonization (which may favour either sensitive or resistant strain). The overall effect of transmission on the frequency of resistance likely depends on the balance of these three effects.

### 4.2. The role of recombination

The rate of recombination between strains colonizing the same host had little effect on linkage disequilibrium, both in a single population (figure 1*d*), and in a structured population (figure 3*c*). This may seem surprising, as recombination is expected to break down LD. In a single population, the most common outcome is the loss of polymorphism and the dominance of a single strain: thus, recombination does not disrupt this equilibrium. In a structured population, polymorphism may be maintained at equilibrium. However, the maintenance of polymorphism implies that the different classes are strongly isolated (low inter-class transmission), and, effectively, distinct strains are rarely in contact in the same host. Thus, recombination also has little effect on the equilibrium.

However, recombination could affect the transient temporal dynamics of the model. In a scenario where the eventual equilibrium is coexistence between sensitive and double resistant strains, recombination accelerates the emergence of double resistance but results in a lower equilibrium frequency of double resistance (electronic supplementary material, figure S5). When the sensitive and two single resistant strains are initially present but the double resistant is initially absent, only recombination between the strains *R*_1_ and *R*_2_ can generate the double resistant strain *R*_12_. Thus, recombination will initially speed up the emergence of double resistance. However, at equilibrium, single resistant strains are constantly generated by recombination between the sensitive and double resistant, resulting in a lower equilibrium frequency of double resistance with increasing recombination. This results in the coexistence of all four possible strains, but only the sensitive and double resistant strains would coexist without recombination.

Previous work showed that the effects of recombination on the transient dynamics of MDR are potentially more complex. For example, when the MDR strain is less costly than expected (negative cost epistasis), the MDR strain is favoured but recombination slows down its spread by creating less fit single resistances [48]. Moreover, recombination is more frequent when the prevalence of the bacterial population in the host is higher. The larger transmission will result in higher prevalence and more recombination, which will affect the transient dynamics of resistance [48]. These processes should take place in our model as well.

## 5. Conclusion

The epidemiology and evolution of resistance to antibiotics depends on the complex ecology of bacteria [18,26,38,47]. Bacteria multiply in a diversity of hosts—already colonized by a strain or not, untreated or treated by a variety of drugs, belonging to different age classes, geographical locations, etc. We formulated an epidemiological-evolutionary simulation model that included these phenomena and elucidated its behaviour with statistical analysis of a large number of simulations and mathematical analysis. We generated a number of predictions, the most interesting of which are (i) multiple single treatments received by the host population as a whole can drive the evolution of MDR; (ii) in a structured host population, the alignment of treatments across classes favours MDR; (iii) transmission and supercolonization favour the evolution of sensitive strains; (iv) higher transmission results in a greater diversity of sensitive and resistant strains. How the alignment of uses of different antibiotics across classes drives MDR has not been empirically investigated so far. To do so, it would be necessary to identify the forms of population structure that are most relevant to bacterial antibioresistance. MDR should occur more frequently for the pairs of drugs with the more aligned uses across classes. More broadly, it would be interesting to link more closely large scale epidemiological datasets on resistance with these new theoretical results.

## Data accessibility

This article has no additional data.
